# ‘System friction’ in China-EU economic relations and the reaction of the EU

**DOI:** 10.1007/s10308-023-00667-9

**Published:** 2023-02-21

**Authors:** Herman Voogsgeerd

**Affiliations:** Departments of International Political Economy (Faculty of Arts) and of Business, European and Tax Law (Faculty of Law), University of Groningen, Groningen, Netherlands

## Abstract

In the period between 2015 and 2020, we have witnessed an increase in ‘system friction’ in the trade and investment relations between the EU and the People’s Republic of China (PRC). This paper focuses on the meaning of this notion of ‘system friction’, originally coined by Sylvia Ostry and on how the EU and especially the European Commission reacted to this friction. This notion might present an alternative to the notion of ‘system rivalry’. The result of system friction in the relation between the EU and the PRC had been a convergence towards more trade defensive moves. A form of managed trade with help of a ratified Investment treaty between the two sides might be a potential outcome.

## Introduction

International trade, long taken for granted in a time of increasing globalisation, has become a sensitive topic during the last decade. The post-war international trade architecture around the General Agreement on Trade and Tariffs (GATT) and the World Trade Organisation (WTO) has led to considerable results in the form of economic growth. Many developing countries, and two very large countries, the People’s Republic of China (PRC) and the Russian Federation, have become members of the WTO.

However, since the demise of the Doha Round, the WTO is apparently in an enduring state of crisis. No major new trade round or major negotiations are scheduled. The dispute settlement system is also in difficulties, as the USA under the Trump administration had started to block the nomination of new members of the Appellate Body (AB). The new administration under President Biden is not ready to radically change the US position on this issue. Large deficits in the trade of goods of the USA with other countries and entities such as China, Japan and the EU have led the US administration under President Trump to question the legitimacy of the WTO system. Even though President Biden has indicated a willingness to be more actively involved in international trade governance, his administration appears determined to address some of the perceived ‘imbalances’ in international trade flows first.

It seems that, from a political perspective, international trade has to be reciprocal, large trade deficits are difficult to sustain over a long period of time. Recently, also political economists such as Dani Rodrik (Rodrik 2018) have questioned the premises of international trade and free trade agreements. Large multinational corporations have become extremely rich because of free trade, but some workers have become poorer or lost their jobs, even in the USA and in parts of Europe. Protests of civil society organisations against mega trade deals such as CETA between Canada and the EU have become louder, and the EU appears to take this kind of protests seriously. After many successful GATT/WTO rounds, trade is increasingly about issues of domestic regulation. Regulatory cooperation is an indispensable part of modern trade deals. This kind of cooperation could lead to the externalisation of regulatory preferences from a large country or bloc such as the EU to a third country.

What are the implications of this changing context for the economic relations between the EU and China? The EU has recently begun to see China as a ‘systemic rival’ that promotes ‘alternative models of governance’ (European Commission 2019). Heads of state such as President Macron of France have also used this kind of terminology. The term ‘rival’ was immediately criticised by China as not being consistent with a positive trade partnership between the two (China Daily 2019). ‘System friction’ between the EU and China, where the role of government is substantially larger, is inevitable in the changing worldwide context, where China has become more powerful economically as well as politically, and where the trade and investment volumes of China in the EU have been increasing over the past two decades. Calls for a ‘level playing field’, whatever this may mean exactly, are becoming stronger. The question is how to deal with such friction between the EU and China in order to prevent system rivalry or even system collapse? I will concentrate on the legal dimension of the relationship and focus on developments in the period 2015–2020, especially on the reaction by the EU to the rise of China as a special kind of competitor by studying some European Commission staff documents and policy plans. The term ‘system friction’ is different from the concept of ‘systemic rival’ and has to be introduced in the next section. The main argument defended here is that ‘system friction’ may function as an alternative to ‘systemic rivalry’. ‘System friction’ may be solved within the institutional framework of the WTO. ‘Systemic rivalry’ in the sense of defending an alternative system of governance is more difficult or even impossible to solve within the WTO context. Both terms are separated here.

## System friction

An important angle from which to study an economic relationship between two countries, or clubs of countries is with the concept of system friction. This term, originally coined by Sylvia Ostry (Ostry 1992 and 1996), presumes that increasing trade relations between countries will lead to several kinds of friction, especially now that tariffs are low and trade is increasingly about regulatory behind-the-border issues. Ostry focuses on those standards related most closely to trade, such as the general legal regime based on transparency, due process and a limited role for the state. In this, the influence of the USA an the beginning of the 1990s is to be seen; US administrative law and its focus on administrative procedures and ‘due process’ has also been influential in the forming of the rules of the GATT/WTO (Ostry 1998, p. 4). In her definition of system friction, two elements prevail: national or sub-national rules that affect market access for foreign companies in a country and, secondly, rules concerning the performance and competitiveness of companies, especially in new sectors and technologies (Ostry a.o., 2000). In 1991, she states that: ‘the battle for market share in leading-edge sectors involves not only competition among multinational enterprises but also rivalry among the different market systems which influence its enterprises’ ability to compete’ (Ostry 1991, p. 83).

These two elements return prominently in the actual economic relationship between the EU and China. The European Union Chamber of Commerce in Shanghai complains regularly about access to the huge Chinese market (*European Union Chamber of Commerce* 2018/2019). Chinese firms on the other hand have acquired access to some important sectors of the European economy. Competition in future technologies such as artificial intelligence is also important in the context of China-EU relations. In this paper, the renewed importance of the term system friction, as an alternative to system rivalry will be argued. This latter term raised a lot of attention and was mentioned in a document from the European Commission and the High Representative of Foreign Affairs only as one in three qualifications of the PRC (European Commission 2019): as a negotiating partner, as an economic competitor especially with respect to technological competition and as a systemic rival. The active promotion of ‘alternative models of governance’ is a core element of the last qualification, and in this sense, it goes beyond the notion of system friction. However, ‘system friction’ captures a crucial element of current tensions in international trade and could be addressed in the context of a reformed WTO. If the EU and China cannot solve ‘system friction’, the free trade system might collapse and the only option will be a kind of managed trade. In this managed trade the overall political relationship between the EU and China will weigh more heavily.

In what policy areas do we find such measures that could cause system friction? Ostry herself mentions competition policy, capital market regulation, social policy, tax law and especially intellectual property law. Ostry’s work is from the beginning of the 1990s, a period that could not be more different from the period since 2015. In the 1990s, the dominant paradigm has been neo-liberalism and the retreat of the state. Ostry presumed in this period the dominance of a common law regime of the Anglo-American type with strong constraints on the role of government (Ostry, 1996, 62-63). She focused on three systems, an Anglo-American, a continental European and a Japanese one. The rise of Japan was very visible and broadly debated in the period in which Ostry wrote her articles.

A quarter of a century later, it is the rise of China that has now reached centre stage in the debate. The difference between the current situation and the 1990s is that geopolitical tensions surrounding the trade regime add much to the friction. I argue that the approach of Ostry on system friction is still applicable in the current context with the rise of China as an economic power and the dominant role of the state and the Communist party (CCP) in this large economy. Due process, transparency and a limited role for the government were important in the 1990s, but also in trade politics in the period 2015–2020. A sufficient degree of ‘convergence’ between standards, laws, implementation and enforcement is needed for frictionless trade (Ostry 1992, p. 4). The question is convergence to what? For Ostry, systems should converge towards ‘that regulatory system that best reflects the preferences of … capital and entrepreneurship’ (Ostry 1992, p. 4). It is not to be expected that this kind of preferences takes centre stage in the current views of the CCP. Whether a global convergence to common and transparent standards is possible in the contemporary situation is doubtful and that is why system friction, if it remains unresolved, may also lead to a second-best option, that is managed trade. External pressure on China worked at a certain moment in the trade negotiations between the USA and China: on 15 March 2019, the Chinese parliament approved a law with the intention to create and improve a transparent environment for foreign firms in China. American and European complaints seemed effective at this specific moment. China appeared to act in the spirit of Deng Xiaoping’s policy of opening up China. More recently, however, the relations between the USA and China deteriorated.

Inevitably, there are power relations involved here. Chad Damro coined the term Market Power Europe suggesting that with its large internal market, the EU would be able to use market power in order to externalise some of its policy preferences (Damro 2012). The Chinese political and economic system is very different from the one dominant in the EU. How will Market Power Europe react to another huge market power defending different norms? System friction manifests itself in different areas and sectors of the economy, especially in relation to market access and competitiveness of firms. Not every inhibition of market access is related to system friction; however, only a substantial involvement of the national or sub-national government or a national regulator will cause problems. For example, in line with Ostry’s own position, I would submit that consumer preferences are not directly related to system friction (Ostry 1991, p. 84). Japanese cars have been very successful in the European market, unlike American brands. Consumer preferences may be constructed over the long term with the help of government intervention, but are not easy to change in the short term. Technological development is also not directly related to system friction. Chinese citizens hardly use credit cards, as payment by mobile phone has been developed fast. Electrical cars are another example where China made real progress. Technological development is also something that can be shaped by governments, just like consumer preferences. Again, only substantial involvement by governments and other state actors in these areas is able to cause system friction between Western and other countries.

Nowadays, regulatory cooperation is an important element in free trade agreements between the EU and other countries around the world. The conclusion of any trade agreement is the starting point for further regulatory cooperation. The important political text *Trade for All* makes an interesting distinction in this respect: regulatory difference may be based on cultural differences and societal choices (European Union 2015, p. 13) and this is acceptable if there is an ‘added safety’ for consumers. If there is no added safety these differences and choices should be qualified as ‘disguised protectionism’. The problem is that there may be divergent views on safety for consumers. We can refer here to the long-standing dispute between the EU and the USA concerning GMOs. Safety for consumers is not only a technical matter, there are emotions involved as well.

Government policies at the national, provincial and local level can cause system friction. Policies related to market access and level playing field between firms on a market belong to important drivers of system friction. These two issues loom largely above the trade and investment relations between the EU and China in the period from 2015 to 2020 studied in this contribution. It is as if Market Power Europe (Damro 2012) is confronted with another Market Power, which forcefully promotes and externalises different market-related policies and regulations. This confrontation is first and foremost a source of anxiety and the idea is getting stronger that the status quo in the relations between the EU and China is unstable and that something has to be done about this. We will see in the next section that the EU is becoming more defensive in its relations with the PRC. Interestingly enough, China’s economic policies are also becoming more defensive in reaction to the strong frictions with the USA over the past few years.

## China-EU economic relations between 2015 and 2020: rising tensions over values and their effect on system friction. From system friction to system rivalry?

What is the role of values that are connected to economic policy in the relations between China and the EU? The EU promotes a so-called ‘responsible trade and investment policy’ with help of new kinds of trade agreements. If we study the EU Commission’s programmatic document on this, *Trade for all* (European Union 2015), some specific values seem to matter increasingly. This attention for values such as respect for labour rights, sustainable development and consumer safety came after protests from civil society against mega trade deals such as CETA with Canada. Civil society actors have become formally involved in the negotiation and follow-up of new trade agreements. Can the non-ratification by the PRC of important core conventions of the International Labour Organisation (ILO) on Freedom of Association and the Right to Organise and Collective Bargaining be qualified as system friction? Here, the relation is, again, indirect, as these core conventions are deemed by the ILO to be ‘global’ values. The EU wants to ‘safeguard’ the European social and regulatory model via bilateral trade agreements (preface Malmström, *Trade for All*). The worst forms of child labour, forced prison labour and corruption are specifically addressed in this *Trade for All* publication. Dialogue with trade partners such as China on these issues is inevitable, but this does not have to directly affect the market access and competitiveness of companies. Only a substantial difference in views on these values between the government and other state actors will lead to complaints concerning the level playing field. The National People’s Congress decided in the beginning of 2022 to ratify the two core conventions on forced labour according to a press release of the ILO from the 20th of April 2022. Even though they relate only indirectly to system friction, values matter a great deal for the overall political relationship between China and the EU. According to article 21, sub b TEU the EU’s external policies are based on values such as ‘democracy, the rule of law, human rights and the principles of international law’. In all new generation of free trade agreements of the EU since the FTA with the Republic of Korea from 2011, these values are essential, although the level of detail of the terminology varies. The values are also addressed in Framework Agreements or Strategic Partnership Agreements that go together with the FTAs the EU concluded with third parties. Implementation and enforcement of the FTAs are another matter of increasing importance for the EU. To say the least, the role of law in China is different from that in the EU. In fact, it seems that the lack of transparency and due process is a particular characteristic of the Chinese system, and that, this is one of the causes for the difficult market access of EU companies in China and the resulting critical voices in Europe on the ‘level playing field’. Different views on values increasingly trickle down in the already existing system friction between the EU and China. Human rights, liberal democracy and freedom are difficult issues in the relationship, Feng Yuan speaks about ideologies in this respect (Yuan 2022, p. 64).

Other values mentioned in article 21 TEU might be less contentious. Banning poverty and helping developing countries (sub d) and protection of the environment (sub f) are examples of these. Ideas on multilateralism and multilateral cooperation (sub h) at first look less contentious, but apparently EU and PRC views increasingly seem to differ on these issues. EU views on multilateralism are more rule-based and Charles Michel, the president of the European Council stated in 2020 that China and the EU do not agree on the kind of multilateralism (quoted in Caffarena and Gabusi, p. 20). The PRC prefers universal instead of Western values (Yuan 2022, p. 71). The value of ‘open trade’ mentioned in article 21, sub e TEU is closely related to the concept of system friction.

System friction is clearly visible in the period under consideration. The European Union Chamber of Commerce in China regularly complains about a lack of market access for European companies. There are complaints about the rule of law, reciprocity and the dominance of State-Owned Enterprises (SOEs) on the Chinese market, including the state of Chinese public procurement laws (*European Union Chamber of Commerce* 2018/2019). This Chamber speaks about a ‘reform deficit’ in China that has to be addressed ‘in order to rebalance China’s trade and investment relationships’ and ‘ease global tensions’. Not only is the Chamber issuing detailed recommendations for China, it is measuring Chinese performance according to ‘globally established norms’ (preface by president Mats Harborn, p. 1). The term ‘level playing field’ is mentioned, as European companies feel that they face more obstacles than Chinese companies on the Chinese market, especially in relation to the many SOEs. The substantial power of Chinese authorities and regulators, also at the provincial and the local level is a major issue of non-transparency and ‘opacity’. In other words, the report of the European Chamber advises the PRC to change the structure and mentality of the Chinese public sector, also to the benefit of Chinese consumers (p. 11). The European Commission also speaks about the need to ‘balance’ trade ties with China (European Commission Press Release, 12 March 2019). This shows concerns about market access and competitiveness of companies, and therefore system friction in Ostry’s sense. Ostry herself is also aware of the importance of a change in the balance of power between states for system friction, she mentions for example the rise of the Global South (Ostry 2006, p. 147).

After dragging on for years, the negotiations between China and the EU in the area of investment rather than trade have led to an agreement at the highest political level in December 2020, the Comprehensive Agreement on Investment (CAI). Ironically, this success has been tainted by disagreements on value-related issues. After the EU decided to impose sanctions on Chinese persons and entities in reaction to the human rights situation in Xinjiang, China reacted by putting several persons in the EU, including some MEPs, on a sanctions list. This in turn led to the suspension of the ratification process of the CAI on 20 May 2021. Taking all this together it illustrates that differences on values and ideologies, even though only indirectly related to the notion of ‘system friction’ which is limited to market access, fair competition and open trade, have had an increasing impact on companies and their operations between the EU and Chinese markets. If this is not dealt with properly, the threshold between system friction on open trade and systemic rivalry is within reach.

## Examples of system friction and EU reactions to it

The first area where the EU reacted to system friction is anti-dumping. Particularly in this area, system friction between the EU and China has become evident. Other measures, that are not particularly targeted to the PRC, will be discussed later. Because of a country report on China and anti-dumping, written by the staff of the European Commission, the new anti-dumping rules seemed to be specifically targeted at China. Traditionally, the anti-dumping policy area has been responsible for many conflicts between the two sides, on topics such as solar panels and electric bikes. Among the main issues in such conflicts has always been the Non-Market Economy Treatment (NMET) of China, which has frequently led to comparatively high anti-dumping duties levied by the EU on Chinese products.

In 2017, the EU introduced a new methodology for calculating the normal value in trade defence situations in Regulation (EU) 2017/2321 amending Regulation 2016/1036 dealing with ‘significant distortions’ in the market of a country exporting to the EU.^1^ This new concept of ‘significant distortions’ replaces the old dichotomy between countries receiving market economy treatment (MET) and those with non-market economy treatment (NMET). It is no surprise that the EU comes with new rules in this respect as article 15d of the Accession Protocol of China to the WTO states that important aspects of NMET expire 15 years after the date of accession, e.g. on 11 December 2016. The term ‘significant distortions’ is a synonym for ‘substantial government intervention’ concerning prices and costs, including the cost of raw materials and energy. This kind of government intervention constrains the free play of market forces, and is an excellent example of Ostry’s system friction as they affect the competitiveness of firms.

The new regulation entered into force in December 2017 and it also demands more evidence on dumping from complainants from the EU. A so-called country report, made by the staff of the Commission, may, however, be used in order to help finding this evidence. The first country report, issued on 20 December 2017, concerns the PRC. The second report from 2020 addresses Russia. Some parts of the report have been underlined by the staff of the European Commission. For reasons of space, I will focus on these parts as they indicate a perception of considerable system friction. The underlined sentences are to be found in part I of the country report, called ‘cross-cutting distortions’. A distortion on a market is not the same as a discrimination, which is often per se illegal, but there may be grounds to look deeper into the cause of the distortions in order to take defensive measures. In anti-dumping cases directed against Chinese companies, the country report has been used extensively, and therefore, it is an authoritative document.

### Serious distortions

The China report is a so-called Commission staff document. The information in it may be used for trade defence purposes including anti-dumping, but it may always be rebutted. The first chapter is focusing on several general characteristics of the Chinese system and focuses on the Chinese constitution, the CCP, the system of 5-year plans at the national and local level, SOEs, the financial system, public procurement and investment restrictions in China (see on three different categories of SOEs Ding 2014). All these elements are related to some of the most obvious examples of system friction, also dealt with in Ostry’s work of the 1990s. However, while access to the Chinese market is an element in general, the all-encompassing active role of the Chinese government and the CCP in the Chinese economy is a *new* element, not discussed by Ostry.

Indeed the underlined words in the section on distortions are related to the role of the state in the economy. The Commission staff for example underlines the following not unusual parts in the 13th Chinese Five-year plan (FYP): ‘we will strengthen the Party’s leadership over legislative work’ and on “improving laws for the socialist market economy’ (p. 11). It underlined part of article 251 of the articles of association of the China Railway Group in which it is stated that the CCP will ‘play the role as the core of leadership, and the political nucleus, and take charge of the direction and overall situation and ensure the implementation of policies’ (p. 29). In the Chinese document ‘Made in China 2025’, the Commission staff puts emphasis on the Chinese will to ‘strengthen overall planning’ and the wish to rely on ‘domestic equipment’ and ‘domestic brands’ (p. 51). The 13th FYP, again, gets attention in 4.2.7. of the Commission report, where the three words ‘guide market behaviour’ and ‘move faster to put in place a new modern industrial system’ are underlined. Even for so-called traditional industries that are not state-owned, China announces that it will ‘transform and upgrade major manufacturing technologies” ‘improve policies to support enterprises’ (p. 57) and ‘encourage mergers and acquisitions’ (p. 58). On p. 71 of the report ‘preferential policies’ are mentioned to encourage certain industry projects above others. The role of the government in the Chinese economy and the direct intervention in the economy by preferring certain projects above others is highlighted as an element showing that in China, things go in a very different manner than in the EU. By implication, the reader is led to suspect that, according to the Commission, a reaction to this behaviour by the Chinese government may be necessary, but what such a reaction might look like is not an element of the country report itself.

Implementation of Chinese plans gets a lot of emphasis in this document and practical examples of implementation are given by the European Commission. Concerning SOEs, the European Commission staff focused on the distinction between article 7 of the Chinese constitution, that ascribes a specific role to SOEs, and the EU treaties, which start from the principle of ownership neutrality (art. 345 TFEU). The Chinese provision makes the pursuit of non-market goals and direct steering by the government not only a possibility, but a certainty. An example is the support of the Chinese State Council and the State-owned Assets Supervision and Administration Commission (SASAC)^2^ for ‘a number of large enterprise groups that are competitive at international level.’ They support the ‘strategic adjustment of the state-owned economy’ and ‘the development of the non-public economy’, and also ‘improve the economic fundamental system allowing the development of the public ownership economy’ (p. 98 and 99).

The role of the state in the credit insurance market is another topic the document is looking at. The corporation Sinosure has a role to play here in accordance with several government policies and the ‘go-abroad’ strategy that had been dominant for much of the last two decades (p. 134). The shadow-banking sector is also introduced. Public procurement is an important sector as well, in which system friction may come to the fore. Here, the lack of precise definitions, large discretionary freedom combined with the possibility to follow other policies is in contrast with EU standards (p. 160). Investment restrictions for foreign corporations are a final topic dealt with. The investment approval processes are cumbersome and detailed (p. 187, 188), and large administrative discretion is a major issue in the Commission document. Again, these are good examples of system friction.

### Production factors in China

The second part of the Staff report deals with distortions in the production factors. The document treats five production factors: land, energy, capital, (raw) materials and labour. Again, the focus is on the role of the state in the economy, preferential policies in order to support specific industries even when they go against WTO rules (p. 224), the large planning toolbox of central and local governments in China (p. 272) and the non-ratification of several fundamental ILO conventions. The conclusion of the second part of the report is that there is ‘significant systemic distortion’ (p. 261) leading to overcapacity and the existence of ‘zombie firms’ (p. 255), and that there are no ‘normal commercial responses’ (p. 260). In this part, the sources used are Chinese government and local government websites (with a case study concerning Hebei province), reports of international organisations as the OECD, the WTO and the International Institute for Sustainable Development and also earlier anti-dumping and anti-subsidy investigations committed by the European Commission itself. Distortions become a worrying phenomenon for a trade relationship when these are severe and substantial.

### Examples of certain sectors in China

The final third part of the report treats distortions in several selected sectoral studies: steel, aluminium, the chemical and the ceramic sector. It focuses on the influence of the government on the foreign operations of Chinese firms and on export patterns (p. 448). The role of the government in China is put in stark contrast to this role under the EU internal market and other rules. Apparently, the role of the government in the economy is the most worrying development and this could underline opaqueness, lack of transparency and due process and therefore be directly relevant to the existence of system friction between the EU and the PRC.

### Application of the new rules and methodology

The new methodology inserted in the basic Regulation 2016/1036 by way of Regulation 2017/2321 has been used for the first time by the European Commission in proceedings against China concerning imports of hot-rolled steel sheet piles after a complaint by European steel corporations and EUROFER.^3^ Already in earlier anti-dumping proceedings against the import of electric bicycles from China, Chinese firms complained that the existence of dumping should be based on prices and costs of the producers in China. This would follow from section 15, subparagraph (a)(ii) of the Protocol of Accession of the PRC to the WTO. The Commission, however, found ‘significant State interference in China’, especially with respect to the aluminium market, and refused Market Economy Treatment. Application of the new methodology shows that the Commission is prepared to act against cases of ‘significant State interference’. The country report is a unique non-binding document as it is particularly targeted against the PRC. Information in this staff document can be rebutted. The report, however, gives a clear overview of system friction between the EU and the PRC more than two decades after the accession of the PRC to the WTO. Anti-dumping measures of the EU are relatively effective to deal with substantial system friction.

### Other EU-measures

Also outside the area of anti-dumping, the EU has taken many new defensive measures, these are deliberately not explicitly focused on the PRC. Two examples are related to the screening of foreign direct investment and to ‘levelling the playing field’ concerning subsidies granted by foreign states (see also European Commission 2017). The first example led to an EU Regulation 2019/452 that entered into force in October 2020 and the second one to a Commission White Paper in June 2020. In both cases, the PRC is not targeted specifically, although it is clear that these texts are a reaction to some practices of Chinese companies. Some of the strong and vague language may also have been copied from other trade partners. Collective security and public order are issues that may trigger the screening of foreign investments through which the EU can protect its essential interests. Both the USA and the PRC increasingly use this kind of security-related terminology, the scope of which is not immediately clear.

Although the screening mechanism of the EU is put in an EU regulation, it is only complementary to already existing screening mechanisms of the member states. The European Commission is not able to block an investment in the EU by a foreign actor, it can only raise concerns. The EU is also carefully looking at WTO rules by referring to article XIV bis (1) (b) and article XIV (a) of the GATS agreement. In those provisions, the right to protect ‘essential security interests’ and ‘public order’ is recognised. The essential interests of the EU need to be taken care of, especially in the case of foreign investors owned or controlled by foreign states. In comparison with anti-dumping measures, this instrument is rather weak, if one compares it for example to the powers of the US President under the rules of the Committee on Foreign Investment in the United States (CFIUS).

The other example, the White Paper of the Commission on foreign subsidies, proposes several ‘redressive measures’ in order to help restore the level playing field. Here, the main target is, again, not the PRC but some of the complaints made regarding to its practices are clearly identified, e.g. the limited openness of its market and lack of transparency and reciprocity in accessing this market and the high level of government interventions in the economy (European Commission 2020, p. 7). A specific Chinese fund to ease Chinese investments in the EU is explicitly mentioned in a reference in this White Paper (European Commission 2020, p. 8, n. 10). In the case of actual or potential distortions because of foreign subsidies, the member states must block investments as a consequence of these subsidies. Therefore, the decision to act is left to the member states. Although these two newer instruments are weak, complementary to the actions of the member states, it has become evident that due process, transparency, reciprocity, level playing field and substantial government interventions in the economy are concerns that clearly point to cases of system friction.

System friction must be addressed and the EU slowly and gradually has tried to do this. System friction is still different from ‘systemic rivalry’. System friction is about market access, fair competition and a level playing field. Issues of system friction may be addressed with the help of defensive measures that are often perfectly admissible under the WTO framework. System rivalry is much more difficult to address within this framework. Promoting alternative systems of governance and an increasing tension about values such as liberal democracy and human rights and even about the notion of multilateral order itself are very difficult to be addressed within the WTO. Developments after the period 2015–2020 under consideration here do not bode well. The aftermath of the corona pandemic may indeed have led to some kind of systemic rivalry on top of system friction. System friction is already asking a lot of energy from the EU and its member states. China continues to be an important trade and investment partner for the EU and a large competitor too. Companies from the PRC are performing increasingly better in comparison to German companies on the EU internal market, as a recent study of the German Economic Institute suggests (IW 2021). So, what is the best way to address this system friction and to prevent a further deterioration in the form of system rivalry? This is the subject of the last section before the conclusion.

## Will mitigation of system friction and the prevention of system rivalry be possible through institutionalized venues?

Traditionally, the WTO has been the main venue for the solution of conflicts coming from system friction. The WTO dispute settlement system has, however, been considerably weakened since the US administration of Trump. We might say that the WTO is in a state of crisis and needs itself to be reformed. How should it deal with member states with a planned economic system with a huge influence of the government that bring overcapacity in its production to other member states’ markets? The new EU anti-dumping rules were immediately criticised. Member states of this international organisation raised concerns to the EU in a meeting of the committee on anti-dumping practices on the 25th of April 2018, where the EU presented its new rules and explained how it would select representative countries.^4^ The concept of ‘significant distortion’ was severely criticised by other WTO members. The EU communicated during the session that it would also apply ‘social and environmental protection’ elements in order to select representative other countries to decide the normal value of a product. Non-ratification of core ILO conventions and multilateral environmental treaties are explicitly mentioned in Regulation 2018/825 in art. 32a, par. 1 that refers to Annex Ia, and the European Commission must show in its annual report how the ILO conventions have been taken into account in the anti-dumping investigations concerning a specific third country. Before the new rules were introduced, China had already filed a complaint on the previous EU rules on anti-dumping (DS516). The dispute settlement in this conflict has been delayed because of a lack of specialised lawyers in the WTO. It was expected in May 2019, but then China asked the panel to suspend its work and as the suspension was not lifted the authority of the panel ended in June 2020.^5^

The position of the EU vis-à-vis the WTO seems to be ambivalent. On the one hand, the WTO and its rule-based order have a central place in the policies of the EU. Bilaterally, China and the EU frequently refer to their shared support for the WTO, and they even have stated their commitment to working together to ‘reform’ the WTO. The WTO should be the centre of the international trade order and should be ‘reinvigorated’ (European Union, *Trade for all* 2015, p. 28-29). On the other hand, the EU with its new generation trade agreements prefers to insert its own ‘values’ in trade agreements such as the promotion of democracy and human rights which will, without doubt, clash with the interests of China. These values will further complicate the system friction that is already present, and this may bring us to system rivalry.

In case the WTO is unable to address basic flaws in world-wide trade relations and long-term trade deficits of for example the USA or the EU, mitigation of system friction is only possible with a form of managed trade. In this respect, a bilateral treaty between the EU and the PRC could become essential. The CAI, however, is suspended at the time of writing of this article. Reciprocity is an important element of the WTO regime, but difficult to operationalise through the DSB. Not only the Trump administration in the USA but also the EU in its communications from March 2019 on the relationship between the EU and China is focused on reciprocity. Reciprocity is well-suited for multilateral negotiation rounds within the framework of the WTO, which have been missing for decades. Now that the Doha Round is defunct, reciprocity has to be dealt with in the framework of bilateral deals between China and the EU such as the CAI. European industry, which is warning against protectionism, is in favour of the ratification of the CAI (ERT 2021).

Recent developments after the period under consideration in this contribution do not send promising signals. Because of the geopolitical conflict with the USA and the aftermath of the Covid-19 pandemic, the PRC in May 2020 changed its economic policy with a new ‘dual circulation strategy’. The PRC is determined to diminish its dependence on foreign markets and its vulnerability to outside pressure and will refocus on its huge domestic market. Only in case of need will foreign products be allowed on the market (Yan Xuetong 2021). This more protectionist industrial policy seems to be a defensive move and may lead to additional complaints by companies from the EU concerning the level playing field. Moreover, there are critical tones in the Chinese press, that there are ‘discriminatory practices’ within the EU against Chinese enterprises (China Daily 2021). It mimics the criticism the European Chamber of Commerce has already voiced for decades on the treatment of European firms on the Chinese domestic market. The ironic aspect in this is that both the PRC and now the EU are reacting in a defensive manner. So, system friction seems to have lead to convergence to more trade defensive moves. If the CAI is not ratified, consensual or coordinated management of trade relations will become more difficult, and conversely bargaining and power will inevitably become more important in bilateral trade relations and by extension in the already severely weakened international trade order. On top comes the covid pandemic. In a policy brief from May 2020, Andrew Small suggests that the handling by the PRC of the origin of the corona virus, and increasing ideological competition instigated through disinformation campaigns, has led to distrust in many capitals of the EU member states of the Chinese government (Small 2020). Restoring trust is therefore essential to stop highly adversarial trends in the relationship between China and the EU (Caffarena and Gabusi 2022, p. 22).

## Conclusion

System friction has been the central notion of this paper. It is inevitable that system friction arises in international trade in the modern era when countries and regions as diverse as the PRC and the EU are all trading under WTO rules. System friction, or ‘substantial distortions’ due to government intervention in the economy, has to be accommodated or managed from a mutually advantageous perspective. In case system friction is not addressed properly, tensions could spiral out of control and possibly lead to system rivalry and system collapse. Sylvia Ostry coined the term system friction in the early 1990s, an era of dominance by the USA with its focus on transparency and due process in international trade, including a focus on shareholder and investor dominance. Neo-liberalism and the retreat of the state were dominant in the context of that period. Although the context is considerably different, the term is applicable to the current state of EU-Chinese economic relations. The preceding analysis has shown many areas where EU discourse and policies towards China demonstrate the importance and effects of system friction in bilateral commercial relations. System friction has to be dealt with. Momentarily, it seems to lead to convergence, albeit not in the direction Ostry preferred, i.e. guided by the preferences and practices of economic agents, nor towards a strengthened rule-based international trade system, but towards a power-based managed trade order in which conflict and cooperation go side by side. Both China and the EU are reacting in a defensive manner. Market Power Europe is confronted with another large market power, the PRC that is not happy with the status quo and prefers ‘universal’ instead of Western norms and values to govern international trade. The best way to accommodate system friction is still within the framework of a ratified investment treaty CAI. This would amount to some sort of coordinated management of economic relations, in which trust between the two sides could slowly grow.

Before the accession of China to the WTO, Rodrik in 1999 stated five (political) principles of international trade. Among other rules, such as not to impose own views on trade partners, or that a country or a group of countries have the right to protect their own societal views, one of these was as follows: do not trade with non-democratic states (Rodrik 1999). It is to be hoped that we do not have to go back to that rule of Rodrik, although the current Czech presidency of the EU (July–December 2022) prefers to conclude trade deals with ‘democratic’ countries. Therefore, the distortions resulting from the large influence of the state, party and government in China have to be addressed. Divergence in values may exacerbate system friction, lead to system collapse or even system rivalry. The focus should lie first, however, on how to deal with the system friction, in order to prevent more system rivalry.

## Footnotes

1 OJ L 338, p. 1, 19 December 2017. Regulation 2016/1036 has subsequently been amended again.

2 SASAC is a commission of the State Council that is responsible for overseeing SOEs, appointing the top directors of the companies and deciding on mergers.

3 OJ, C 177/6, 24 May 2018. The staff report has been used in several anti-dumping cases recently.

4 https://www.wto.org/english/news_e/news18_e/anti_25apr18_e.htm, (accessed 21 March 2022).

5 It seems that the result of the procedure may have been unfavourable to China and as the operation of the AB was blocked by the US administration the panel would have been the ultimate outcome of the DSB. (https://ielp.worldtrade.net/2019/06/china-nme-case-suspended.html, accessed 25 May 2021).
